# Various Strategies in Post-Polymerization Functionalization of Organic Polymer-Based Monoliths Used in Liquid Phase Separation Techniques

**DOI:** 10.3390/molecules25061323

**Published:** 2020-03-13

**Authors:** Sarah Alharthi, Ziad El Rassi

**Affiliations:** 1 Department of Chemistry, Taif University, Taif 21944, Saudi Arabia; sarah.alharthi@tu.edu.sa; 2 Department of Chemistry, Oklahoma State University, Stillwater, OK 74078, USA

**Keywords:** organic polymer monoliths, post-polymerization functionalization, HPLC, capillary LC, capillary electrochromatography

## Abstract

This review article is aimed at summarizing the various strategies that have been developed so far for post-polymerization functionalization (PPF) of organic polymer-based monoliths used in liquid phase separation techniques, namely HPLC at all scales and capillary electrochromatography (CEC). The reader will find the organic reactions performed on monolithic columns for grafting the chromatographic ligands needed for solving the separation problems on hand. This process involves therefore the fabrication of template monoliths that carry reactive functional groups to which chromatographic ligands can be covalently attached in a post-polymerization kind of approach. That is, the template monolith that has been optimized in terms of pore structure and other morphology can be readily modified and tailor made on column to fit a particular separation. The review article will not only cover the various strategies developed so far but also describe their separation applications. To the best of our knowledge, this review article will be the first of its kind.

## 1. Introduction 

In principle, the simplest approach for making monolithic column involves the direct co-polymerization of functional monomers with crosslinkers in order to yield monolithic stationary phases with the desired functionalities. Although this direct approach may look at a first glance simple and can be readily achieved, the choice of porogens, polymerization temperature and other conditions still involve many trial and error to reach an optimum monolith. Therefore, time consuming and labor-intensive re-optimization of the polymerization conditions from one monolith to another is required to get a monolith with satisfactory chromatographic performances. In addition, polar and nonpolar functional monomers are not always directly and/or easily available and some monomers are incompatible with certain co-polymerization processes. As an alternative route of monolithic column preparation, post polymerization functionalization (PPF) of precursor monoliths allows the introduction of various polar and nonpolar ligands onto the monolithic surfaces starting from one generic and optimized template monolith. In that sense, PPF is an essential technique used to modify the monolithic surface chemistry for use in a variety of studies. Furthermore, the major advantage of using PPF is that a variety of columns with different retentive properties can be obtained from one generic optimized monolith matrix. So far, various PPF procedures have been described and demonstrated in the literature to yield separation performances in different separation methods, such as capillary electrochromatography (CEC), HPLC, capillary LC and nano-LC. Thus, it is the aim of this review article to summarize the various strategies in PPF of monoliths and demonstrates that the PPF is an efficient process for fast development in stationary phases. In this review article, the different template monoliths which have been applied in PPF, will be divided into precursor monoliths with epoxy, succinimide, hydroxy, chloro and carboxy functionalities as well as those based on photografting, hypercrosslinking, incorporated nanoparticles and miscellaneous processes. The various PPF strategies performed on the different precursor monoliths have allowed the efficient separations of different analytes using a few original monoliths as the starting columns, which helped to save time and consumable.

## 2. Various PPF Strategies 

### 2.1. Monoliths with GMA Functional Monomers 

**Scheme 1:**
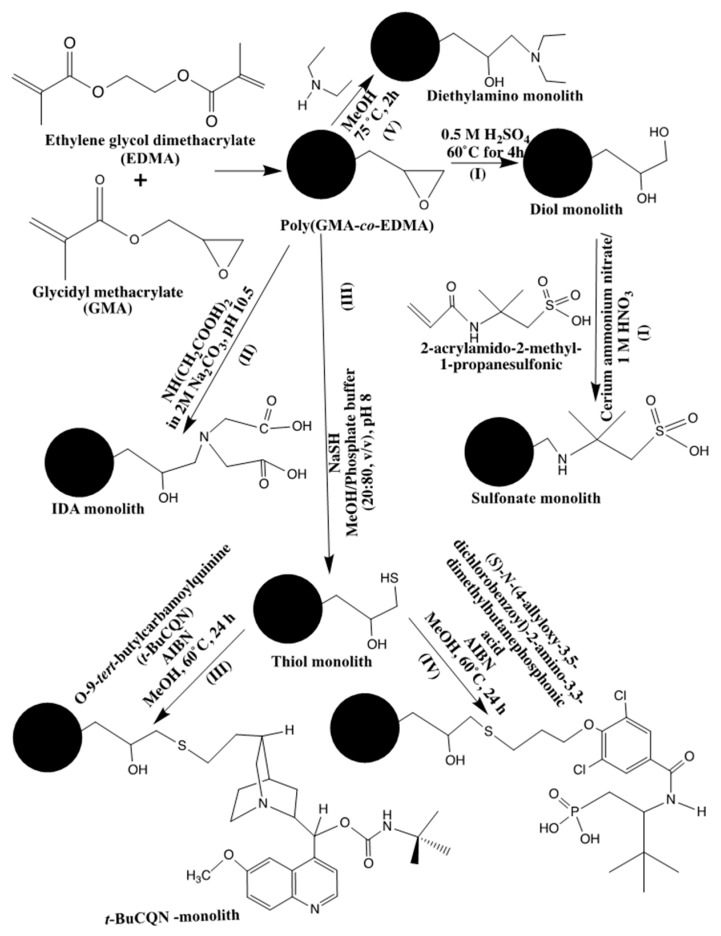
Routes I through V for post polymerization functionalization (PPF) of poly(glycidyl methacrylate (GMA)-based monolith.

poly(glycidyl methacrylate (GMA)-*co*-ethylene dimethacrylate (EDMA)) monoliths grafted with poly(2-acrylamido-2-methyl-1-propanesulfonic acid) chains were prepared and used for the separation of proteins by ion-exchange HPLC mode [1]. The precursor monolith was synthesized via in situ polymerization of a mixture consisting of GMA as functional monomer and EDMA as cross-linker in a binary porogenic solvent containing cyclohexanol and dodecanol in the presence of AIBN as the thermal initiator. This was followed by converting the epoxy monolith to a diol monolith using acid hydrolysis at 60 ˚C (route I in Scheme 1). The diol monolith thus obtained constituted the substrate onto which a grafting can be performed via the transformation of the surface hydroxyl groups to free radicals by the Ce(IV) initiation which yield a single radical on the oxygen atom of the hydroxyl group according to the following simplified mechanism:

Ce(IV) + RCH_2_OH → Ce(III) + H^+^ + RCH_2_O.

The free radicals allowed the grafting of poly(2-acrylamido-2-methyl-1-propanesulfonic acid) onto the internal surface of the porous diol monolith (see route I in Scheme 1). The optimization of the experimental conditions was investigated, and the study demonstrated that the method successfully separated three proteins (i.e., myoglobin, chymotrypsinogen A and lysozyme) within 1.5 min. 

Recently, PPF allowed the covalent attachment of iminodiacetic acid (IDA) to a poly(GMA-*co*-EDMA) continuous rod column [2]. In this case, the precursor monolith was prepared via in situ polymerization of a mixture containing GMA and EDMA in the presence of a binary porogenic mixture of dodecyl alcohol and cyclohexanol under thermal initiation condition. The continuous rod column thus obtained was then subjected to PPF with IDA (see route II in Scheme 1) and was used in immobilized metal affinity chromatography (IMAC) with IDA chelated with a bivalent metal ion, e.g., Cu^2+^, Ni^2+^ or Zn^2+^. The monolithic rod in the form of naked IDA or Cu^2+^-IDA confined in a stainless steel tubing of 5 cm × 4 mm I.D. allowed the purification of lysozyme from egg white and human serum albumin (HSA) from the commercially available HSA solution as was confirmed by matrix-assisted laser desorption-ionization time-of-flight mass spectrometry. 

In a rather complex PPF process, a poly(GMA-*co*-EDMA) monolithic capillary was first modified with sodium hydrogen sulfide to obtain a monolith with reactive thiol groups whereby about 17% of the total epoxide moieties of the monolithic precursor were converted to thiol as was determined by a disulfide-exchange reaction [3]. In a further PPF step, a quinine derivative was attached to the thiol activated monolith and the column thus obtained was assayed by CEC in the separation of the enantiomers of *N*-3,5-dinitrobenzoyl-leucine (see Route III in Scheme 1). Using the thiol activation of poly(GMA-*co*-EDMA) monolith, the resulting monolith was further functionalized with (*S*)-*N*-(4-allyloxy-3,5-dichlorobenzoyl)-2-amino-3,3-dimethylbutanephosphonic acid as chiral selector to yield enantioselective strong cation exchanger (SCX) capillary columns of 100 µm I.D. (see Route IV in Scheme 1). However, and due to the lipophilic character of the poly(GMA-*co*-EDMA), the enantioseparations were not satisfactory. In order to overcome the lipophilicity of the precursor monolith, 2-hydroxyethyl methacrylate (HEMA) monomer was co-polymerized with GMA and EDMA leading to the poly(GMA-*co-*HEMA-*co*-EDMA) precursor of less lipophilicity, which then allowed as good enantioseparations as those with silica-based enantioselective SCX by CEC [4]. 

**Scheme 2:**
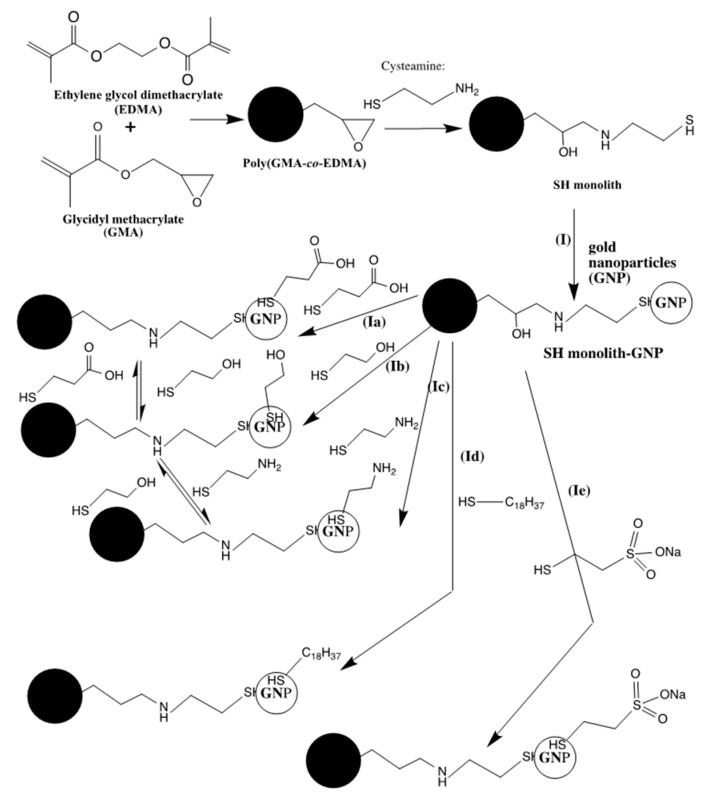
Routes for immobilizing gold nanoparticles onto GMA-based monoliths and the subsequent attachments of polar and nonpolar ligands.

A monolithic capillary column made of poly(GMA-*co*-EDMA) and coated with gold nanoparticles (GNP) was suggested for proteomics analysis that would enable the capture and separation of cysteine containing peptides [5]. The precursor poly(GMA-*co*-EDMA) monolith was subjected to PPF with both sodium hydrogen sulfide and cysteamine in separate experiments to obtain thiol modified monoliths. The resulted thiol monolithic columns were modified with GNP by passing chloroauric acid within the columns (see route I in Scheme 2). Due to their high affinity toward gold, cysteine containing peptides were effectively and selectively captured by the GNP-modified monoliths. In a continuation set of studies, the same thiol activated precursor poly(GMA-*co*-EDMA) monolith was loaded with colloidal GNP by pumping the suspension through the column (see also route I in Scheme 2). This approach afforded higher surface coverage with GNP than the in situ reduction of chloroauric acid within the column. This type of monolith with bound GNP permit the functionalization of the surface with low molecular weight thiol-containing surface ligands (see routes Ia, Ib, Ic, Id and Ie in Scheme 2). Thus, a single monolithic column would be used in different separation modes. These columns with exchangeable chemistries were demonstrated in CEC, micro- and nano-HPLC separations of peptides and proteins in both RPC and ion exchange modes [6]. 

The preparation of methacrylate-based diol monoliths for the separation of polar and nonpolar solutes under hydrophilic interaction liquid chromatography (HILIC) and reversed-phase chromatography (RPC) modes in capillary LC was reported [7]. In this work, after copolymerizing GMA and poly (ethylene glycol) dimethacrylate (PEGDMA) in situ in a 150 mm × 320 µm I.D. capillary column, the obtained monolith was flushed with sulfuric acid solution to form diols at the surface of the monolith. The results showed that increasing the GMA content in the monolith led to increasing the monolith hydrophilicity. The poly(GMA-*co*-PEGDMA) monolith was evaluated in the separation of phenol compounds, phthalic acids and polyaromatic hydrocarbons (PAHs). Commonly, the critical compositions of the mobile phases corresponding to the transition from the HILIC to the RPC mode were around 70% acetonitrile in water. The separation of polar analytes, such as phenols and amides, was commonly performed with more than 90% acetonitrile content in the mobile phase to achieve good retention. The critical value of the poly(GMA-*co*-PEGDMA) monolith stationary phase was gained when the content of acetonitrile in the mobile phase exceeded 90%, and for separation of phenols 98% acetonitrile in the mobile phase was needed. This result indicated that this monolith still has low hydrophilicity.

In terms of applications of monolith precursors subjected to PPF processes one can cite a poly(GMA-*co*-EDMA) monolithic that was converted to an anion exchange column via PPF with quaternary amine groups through the reaction of the monolith with trimethylamine. The resulting anion-exchange column proved useful for the determination of the low level of bromate in food and drinking water by ion chromatography [8]. The precursor of the anion exchange monolith was based on poly(GMA-*co*-EDMA) prepared via in situ polymerization inside a stainless steel tubing. This method successfully detected bromate after the post reaction and showed good LOD, linearity, short analysis time of 8.5 min and reproducibility. Another application involved the separation of DNA fragments and oligonucleotides on a poly(GMA-*co*-EDMA) precursor monolith prepared in a microbore column that was converted to a weak anion-exchange monolithic bearing diethyl amine ligands via PPF (see route V in Scheme 1) [9]. The prepared monolith demonstrated good potential for the separation of DNA molecules and oligodeoxythymidylic acids and can be used for the rapid separation of biomolecules as well as in various applications in biomedical and pharmaceutical analysis. 

### 2.2. PPF Based on Photografting

**Scheme 3:**
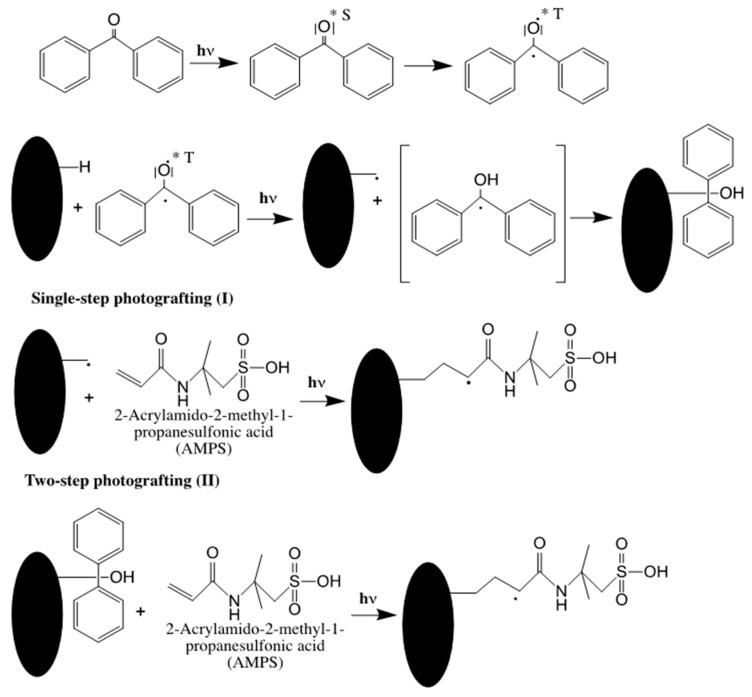
Routes I and II for surface photografting. Adapted with modification [13].

Surface photografting is an efficient method for the modification of polymer surfaces, invented and developed in the laboratories of Ranby and first published in 1986 by Ranby et al. [10]. This process has recently gained popularity in monolith fabrication. For instance, the separation of metal cations was achieved using IDA functionalized organic polymer monolith by capillary high- performance immobilized metal chelate chromatography [11]. In this work a poly (lauryl methacrylate (LMA)-*co*-EDMA) monolith was prepared in UV-transparent fused silica capillary followed by photografting of a poly(GMA) layer on the monolith surface through the benzophenone activated surface following a similar route to that in Scheme 3, (see route II). The difference resides in substituting GMA to 2-acrylamido-2-methyl-1-propanesulfonic acid (AMPS). The grafted monolith was then modified with IDA through the ring opening of the epoxy on the GMA structure according to Route II shown in Scheme 1. The results demonstrated that increasing the concentration of GMA led to an increase in the retention factors of analytes. The proposed monolith was applied to the separation of alkaline earth and transition metal ions in natural and potable waters. 

**Scheme 4:**
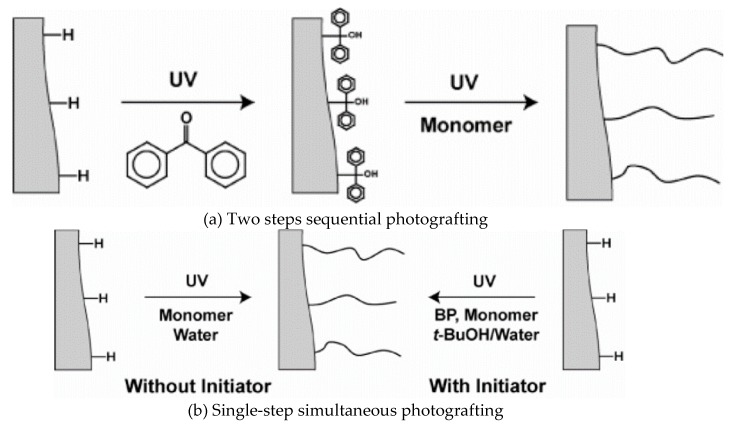
Scheme of the (**b**) single- and (**a**) two-step photografting procedures. Taken with permission from [12].

The photografting of a monolithic capillary column prepared by the in situ co-polymerization of butyl methacrylate (BMA) and EDMA in a binary porogen consisting of 1-decanol and cyclohexanol in the presence of the initiator 2,2-dimethoxy-2-phenylacetophenone (DMPA) has been reported [12]. The prepared monolithic capillaries were subjected to PPF in single- and two-step photografting techniques in the aim of reducing protein adsorption to the monolith of interest (see Scheme 4). In the single step photografting, there are two procedures, the original monolith was flushed with monomer solution without initiator and the other way was to flush the original monolith with monomer solution with initiator and both were exposed to UV irradiation (see Scheme 4). The two-step photografting was obtained by flushing the original monolith first with only initiator and exposed it to UV irradiation, then the obtained monolith was flushed with monomer solution and exposed again to UV irradiation. The adsorption of protein was reduced to less than 2% when using PPF compared to unmodified monolith surface. It has been shown that the single-step without initiator and two-step techniques were more effective than the single-step with initiator. The authors expected that the proposed monolith can be used for multifunctional microanalytical devices.

Furthermore, PPF via a photografting process has been used to prepare a monolithic capillary for the separation of intact proteins and peptides in two-dimensional liquid chromatography coupled with mass spectrometry [13]. The precursor monolith was prepared via UV photopolymerization of a mixture of BMA and EDMA using cyclohexanol and 1-dodecanol as the porogenic solvents. After polymerization, the monomer 2-acrylamido-2-methyl-1-propanesulfonic acid was grafted to the BMA-*co*-EDMA monolithic capillary in single-step and two-step photografting as described previously to yield a strong cation exchange stationary phase, see Scheme 3. The results showed the successful separation of 11 intact proteins that were obtained within 20 min by implementing a gradient across a limited RPC composition window in the second dimension. 

### 2.3. PPF of Hypercrosslinked Monoliths 

**Scheme 5:**
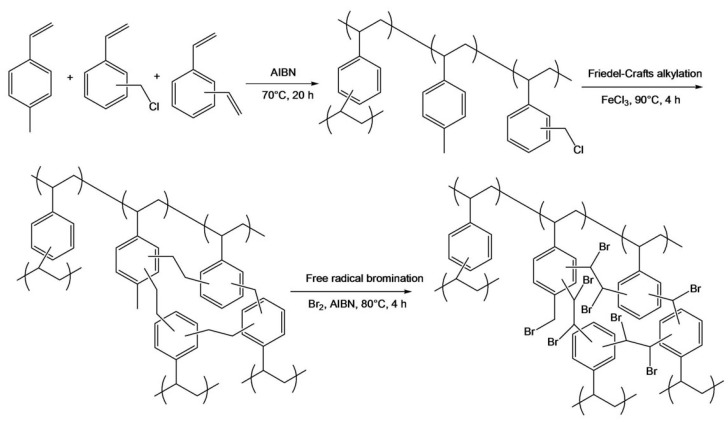
Scheme of preparation of poly(4-methylstyrene-*co*-vinylbenzene chloride-*co*-divinylbenzene) monolith, its hypercrosslinking and free radical bromination with molecular bromine. Taken with permission from [14].

**Scheme 6:**
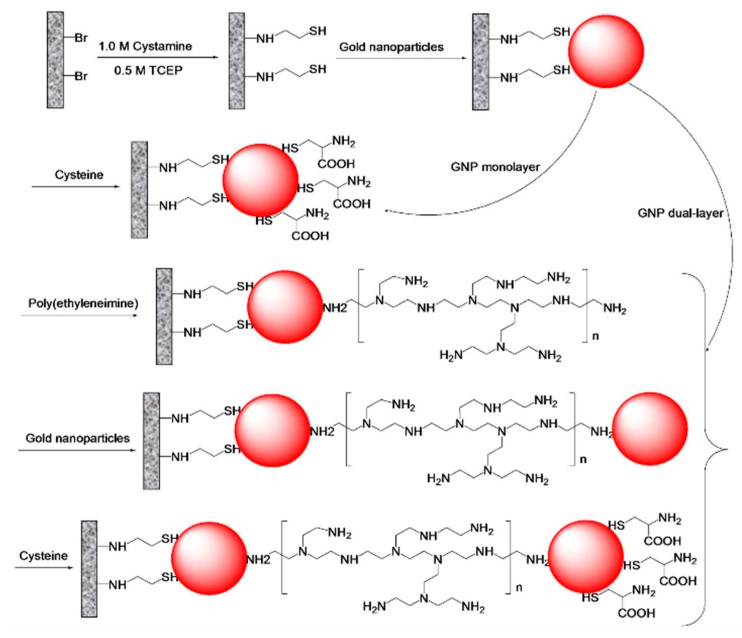
Scheme of modifications of brominated hypercrosslinked poly(4-methylstyrene-*co*-vinylbnezene chloride-*co*-divinylbenzene) monolith with cystamine, tris(2-carboxylethyl) phosphine (TCEP), gold nanoparticles, polyethyleneimine and cysteine. Taken with permission from [14].

Hypercrosslinked porous polymer monolithic capillary columns modified with zwitterionic functionalities were developed through the interaction with GNPs in a layered structure for performing separations using the HILIC mode [14]. The proposed monolithic capillary column started with a preparation of a generic hypercrosslinked poly(4-methylstyrene-*co*-vinylbenzyl chloride- *co*-divinylbenzene) via a Friedel–Crafts reaction catalyzed with iron chloride to achieve large surface area of 430 m^2^/g, see Scheme 5. Then, additional surface functionalization using a free radical bromination was obtained to enable their modification as shown in Scheme 5. Afterwards, the brominated monolith was reacted with cystamine under microwave irradiation for 30 min and reached the maximum of sulfur content. The disulfide bond was snipped using tris(2-carboxylethyl) phosphine (TCEP) to liberate the thiol groups. The next step was to pump the GNPs through the monolith to obtain the first layer of GNP-functionalized monolith. To further increase the column efficiency, polyethyleneimine (PEI) was attached onto the GNP-functionalized monolith followed by a second layer of GNPs that were then functionalized with zwitterionic cysteine, see Scheme 6. This proposed monolith showed “thick” hydrophilic layer using the dual-layer approach with GNPs that included PEI and cysteine functionalities and was tested with the separation of nucleosides and peptides with separation efficiency of 51,000 plates/m at a flow rate of 0.3 µL. 

**Scheme 7:**
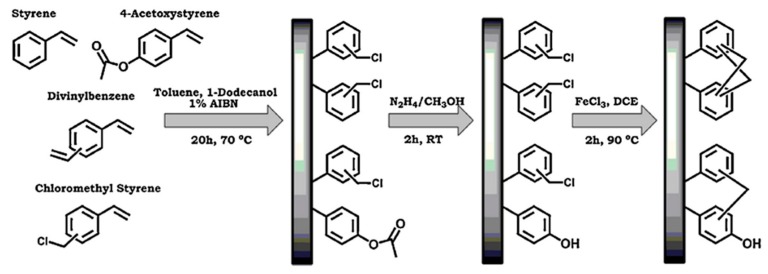
Simplified scheme showing the preparation of hypercrosslinked monoliths with phenol functionalities using copolymerization of 4-acetoxystyrene, followed by deprotection and hypercrosslinking. Taken with permission from [15].

The preparation of porous polymer monolith possessing both large surface area and functional groups for nano-LC was developed [15]. First, the generic monolith was prepared in a capillary by the in situ thermal polymerization of a mixture containing 4-acetoxystyrene, styrene, vinylbenzyl chloride, divinylbenzene and porogenic solvents that consisted of toluene and 1-dodecanol (see Scheme 7). After the polymerization was completed, a solution of hydrazine hydrate was pumped through the monolithic capillary to obtain hydroxyl functional groups resulting from the deprotection of the acetoxy groups. The post-polymerization hypercrosslinking of the monolith was then obtained by adding ferric chloride to offer a large number of small pores thus enhancing the surface area (see Scheme 7). The resulting monolith was evaluated in the separation of small molecules using RPC and normal phase chromatography modes. A column efficiency of 40,000 plates/m was obtained for benzene in the RPC mode, while 31,800 plates/m was achieved for nitrobenzene in the normal phase mode. 

**Figure 1:**
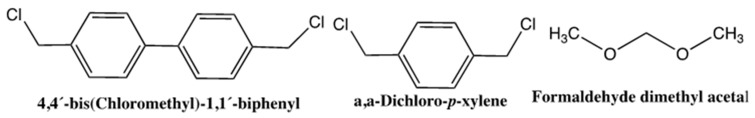
The structure of 4,4’-bis(chloromethyl)-1,1´-bibhenyl, α-α´-dichloro-p-xylene and formaldehyde dimethyl acetal [16].

The same research group developed a new approach of the preparation of large surface area monolithic capillary columns using external crosslinkers for the separation of small molecules in nano-LC [16]. The poly(styrene-*co*-divinylbenzene) monolith was prepared by an in situ thermal polymerization process followed by a hypercrosslinking process using the catalyst FeCl_3_ under Friedel–Crafts reaction. The reaction process included three external crosslinkers, such as 4,4’-bis(chloromethyl)-1,1´-bibhenyl, α-α´-dichloro-p-xylene and formaldehyde dimethyl acetal, see the following structures (Figure 1). The results revealed that the 4,4´-bis(chloromethyl)-1,1´-bibhenyl crosslinker represented the best separation performance and large surface area that reached 900 m^2^/g. The hypercrosslinked monolithic capillary columns were tested in the separation of alkylbenzenes by RPC and the column efficiencies were found to be higher than 70,000 plates/m. 

**Scheme 8:**
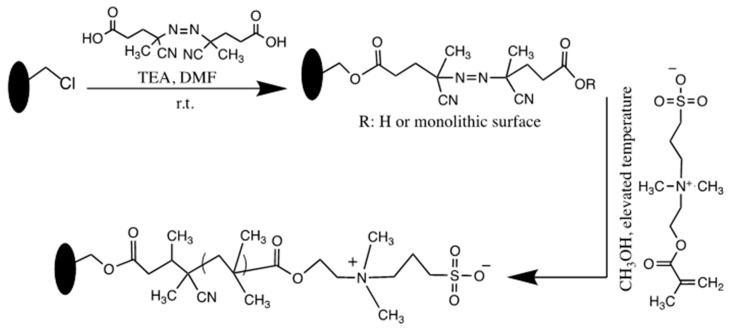
Scheme showing the activation of hypercrosslinked stationary phase together with thermally initiated surface grafting of zwitterion functional monomer. Adapted with modification [17].

The high stability of post-polymerization hypercrosslinking modification of monolithic capillary columns in HILIC was investigated [17]. A poly(styrene-*co*-vinylbenzyl chloride-*co*-divinylbenzene) monolith was prepared by an in situ thermal polymerization. The monolithic surface was then subjected to PPF with 4,4´-azobis(4-cyanovaleric acid) followed by a surface grafting polymerization of dimethyl(3-sulfopropyl)ammonium hydroxide to obtain hypercrosslinked monolith, see Scheme 8. The resulting monolith was used for the separation of small polar compounds in the HILIC mode. The results showed that the grafting time is the major variable for controlling the retention of analytes. The columns showed a dual retention mechanism, including RP and HILIC. The stability and the long lifetime of the columns were obvious. The hypercrosslinked monolith was also used for the separation of polar phenolic acids in the HILIC, 1- and 2D LC. 

The preparation of a hypercrosslinked monolith under nucleophilic substitution was developed for RPC and HILIC using capillary LC [18]. The poly(styrene-*co*-vinylbenzyl chloride-*co*-divinylbenzene) monolith was prepared by in situ radical polymerization. The diaminoalkanes (e.g., 1,2-diaminoethane, 1,4-diaminobutane, 1,6-diaminohexane and 1,8-diaminooctane) were used in the hypercrosslinking process of the monolith through the nucleophilic substitution reaction. The column efficiency was improved when using the longest diaminoalkane with 1,8-diaminooctane. In order to improve the monolith for use in HILIC mode, the hypercrosslinked monolith was further modified with 2-aminoethanesulfonic acid. This proposed monolith provided dual-retention mechanism involving RPC and HILIC. The separation of low-molecular phenolic acids in both RPC and HILIC modes was demonstrated and the hypercrosslinking led to a significant separation performance of alkylbenzenes in RPC.

### 2.4. PPF Involving Click Coupling of Monoliths

**Scheme 9:**
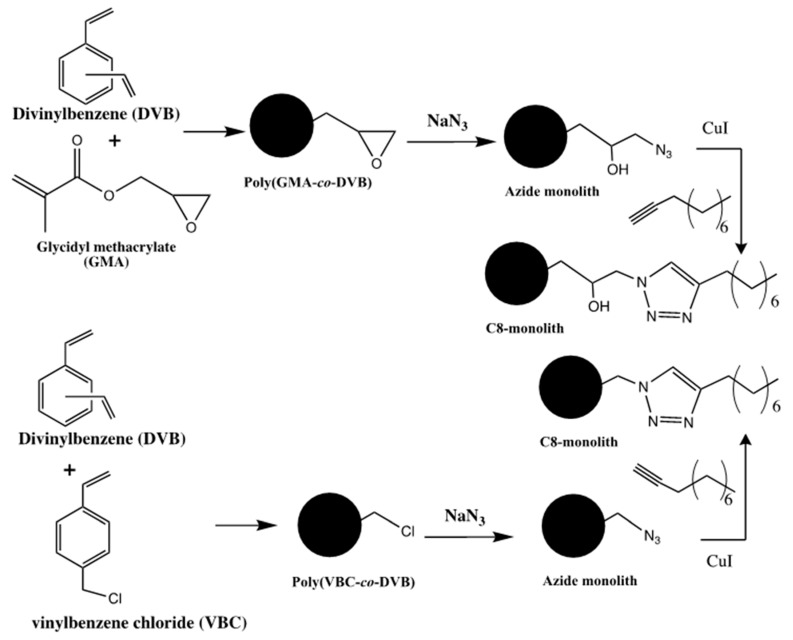
Click coupling of 1-decyne to poly(GMA-*co*- divinylbenzene (DVB)) and poly(4-vinylbenzyl chloride (VBC)-*co*-DVB) monoliths. Adapted with modification [19].

In a study focused on the separation of proteins, poly(GMA-*co*-divinylbenzene (DVB)) and poly(4-vinylbenzyl chloride (VBC)-*co*-DVB) were prepared and then modified with azide functionalities to achieve the reactive sites for a subsequent click chemistry [19]. The azide modified monoliths were then subjected to PPF with a solution of 1-decyne and CuI to obtain alkylated monoliths via the application of copper(I)-catalyzed (3 + 2) azide-alkyne cycloaddition, see Scheme 9. The chromatographic performances of the “clicked” stationary phases were demonstrated with the high separation efficiency for a variety of proteins within 4 min.

**Scheme 10:**
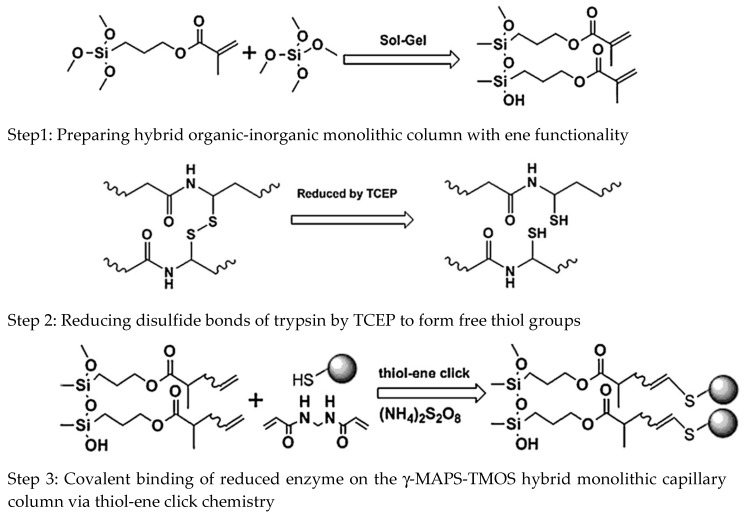
Preparation strategy of trypsin microreactor via thiol-ene click chemistry. Taken with permission from [20].

A novel thiol-ene “click” strategy has been applied for the preparation of hybrid organic-inorganic monolith-based trypsin microreactor [20]. The preparation of hybrid organic-inorganic monolithic columns consisted of tetramethoxysilane and *γ*-methacryloxypropyltrimethoxysilane. Then, the trypsin immobilization solution, which contained free thiol groups, was passed onto the obtained capillary and then the capillary was kept at 25 °C for 5 h to form trypsin microreactor, see Scheme 10. The study involved on-line capillary zone electrophoresis and the results showed that the trypsin microreactor could give highest activity with poly(*N*,*N*′-methylenebisacrylamide). The thiol-ene “click” strategy is a promising method for the selective immobilization of proteins. 

**Scheme 11:**
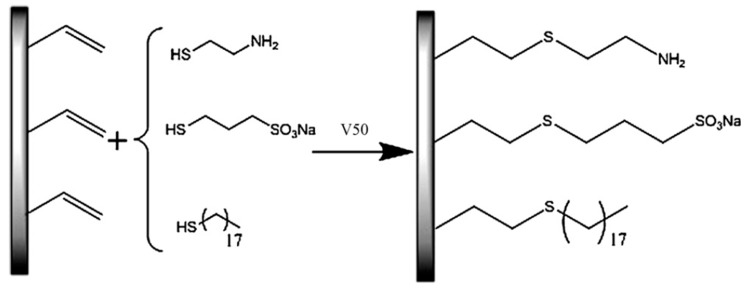
Scheme for the modification of the vinyltrimethoxysilane (VTMS) hybrid monolith with functional monomer compounds. Taken with permission from [21].

An organic-inorganic hybrid silica monolithic column containing n-octadecanethiol (C_18_), 3-mercapto-1-propane-sulfonate (MPS), and vinyltrimethoxysilane (VTMS) has been prepared through thiol-ene “click” strategy (Scheme 11) for CEC [21]. The obtained monolithic column showed high hydrophilic property compared to unmodified VTMS monolithic column. The proposed column can also be used in different separation modes, such as RPC. It showed successful separations of benzenes, aromatic amines, acids and peptides. 

**Scheme 12:**
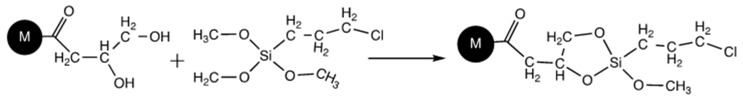
Generation of chlorine functionality into the acid hydrolyzed monolith. Adapted with modification [22].

**Scheme 13:**
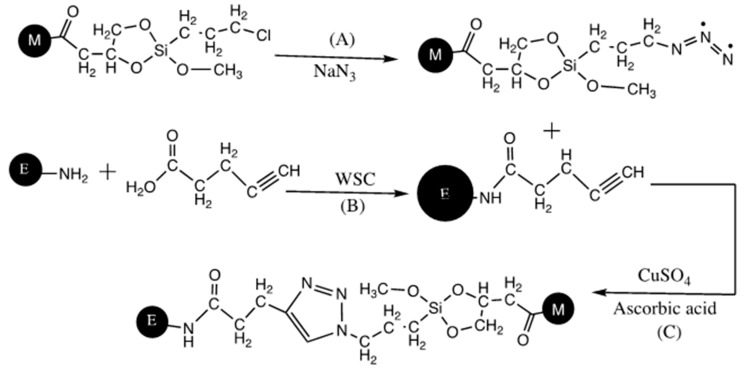
Schemes for the attachment of alkyne-functionalized enzyme onto the azide-functionalized monolith via triazole ring formation based on click-chemistry. (A) Azide formation on the monolith, (B) water-soluble carbodiimide activation and (C) covalent attachment of α-chymotrypsin (CT) by triazole ring formation via click chemistry. Adapted with modification [22].

The preparation of a monolithic-immobilized enzyme reactor by click-chemistry for micro-LC was recently reported [22]. It involved the preparation of a poly(GMA-*co*-EDMA) monolith via in situ thermal polymerization in capillary format. The epoxypropyl groups of monolith were converted to diol form by a well-established acidic hydrolysis process. Terminal chlorine moiety was obtained on the monolith by following a silane coupling chemistry based on the reaction between trimethoxysilyl groups of the reagent 3-chloropropyltrimethoxysilane (CPTMS), and diol groups of hydrolyzed monolith (Scheme 12). NaN_3_ was then reacted with terminal chlorine of the monolith to generate azide functionality (Scheme 13). On the other hand, alkyne-carrying enzyme was obtained by reacting an alkyne-functionalized ligand, pentynoic acid (PA) with α-chymotrypsin (CT) via amide bond formation between carboxyl group of PA and amine groups of enzyme via water-soluble carbodiimide activation (Scheme 13B). In the last stage, the alkyne-carrying enzyme was covalently attached onto azide-functionalized monolith via triazole ring formation according to click-coupling reaction (Scheme 13C). The obtained monolithic capillary column was then modified with the enzyme α-chymotrypsin through the covalent attachment by triazole-ring formation. The activity behavior of the proposed monolith was studied using benzoyl-L-tyrosine ethyl ester as the synthetic substrate. The results revealed that the final substrate conversion increased with increasing the feed flow rate and substrate feed concentration due to the increase of the interaction between the substrate and the enzymatic monolith reactor. 

**Scheme 14:**
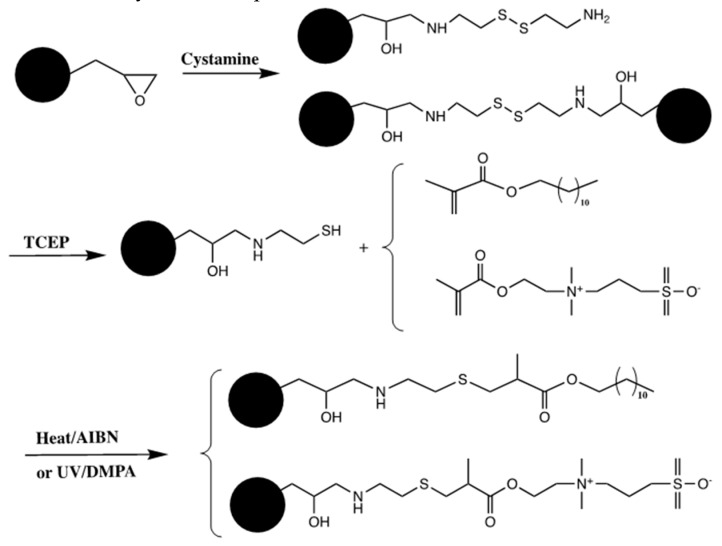
Reaction scheme for the preparation of monoliths and their functionalization via thiol-ene click reaction. Adapted with modification [23].

The preparation of porous polymer monoliths with dodecyl and zwitterionic functionalities via “thiol-ene” click chemistry for the chromatographic separations in RPC and HILIC modes was studied [23]. The generic monolithic capillary columns were prepared via in situ polymerization of a mixture containing GMA and EDMA in the presence of porogenic mixture of cyclohexanol and 1-dodecanol under thermal initiation conditions. The obtained poly(GMA-*co*-EDMA) was subjected to PPF with cystamine to obtain high sulfur content surface. Finally, the unreacted epoxy groups were capped either with propylamine for a hydrophobic monolith or with ethanolamine for a hydrophilic monolith. The next step involved passing tris(2-carboxylethyl)phosphine hydrochloride solution through the column to obtain thiol monolith, see Scheme 14. Then, the hydrophobic and hydrophilic monoliths were prepared as follows. The hydrophobic monolith was prepared by passing LMA through the monolithic capillary column, while zwitterionic dimethyl-(3-sulfopropyl) ammonium betaine (SPE) was used for the preparation of hydrophilic monolith under both photoinitiation and thermal initiation approaches. The excellent performance of dodecyl monolith was obtained by separating three proteins in less than 8 min. Another proof of successful functionalization with long alkyl chains was measured with the retention factor of amylbenzene, which increased by 16 folds after the functionalization with LMA and the column efficiency was increased from 3000 plates/m to 20,000 plates/m. In contrast, the hydrophilic monolith with SPE showed separation of peptides and nucleosides with an efficiency of 19,000 plates/m. 

**Scheme 15:**
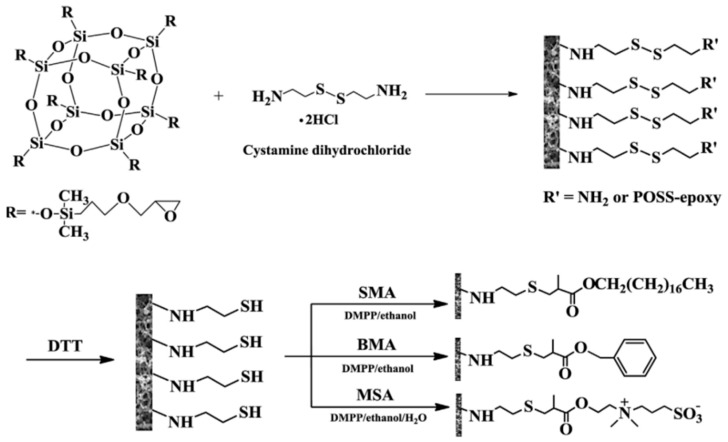
Preparation of polyhedral oligomeric silsesquioxanes (POSS)-based hybrid monolith via ring opening polymerization and post-functionalization based on thiol-ene click reaction. Taken with permission from [24].

Polyhedral oligomeric silsesquioxanes (POSS)-based hybrid monolith was also developed by thiol-ene click reaction for the use in RPC and HILIC [24]. The precursor monolith was obtained via in situ polymerization of a mixture of octaglycidyldimethylsilyl POSS, cystamine dihydrochloride, cetyltrimethyl ammonium bromide and ethanol (Scheme 15). After polymerization, the reduction of disulfide bonds was performed with dithiothreitol (DTT), followed by modification of hybrid monolithic columns in two steps via thiol-ene click chemistry. Stearyl methylacrylate and benzyl methacrylate were chosen to functionalize the hybrid monolithic columns for RPC, while dimethyl-(3-sulfopropyl)-ammonium hydroxide was selected to modify the hybrid monolithic column for HILIC. These proposed hybrid monolithic columns can be successfully used for the separation of small molecules with high efficiency and long hybrid monolithic columns were also prepared to achieve the separation of complex biosamples.

### 2.5. PPF of NAS Based Monoliths 

**Scheme 16:**
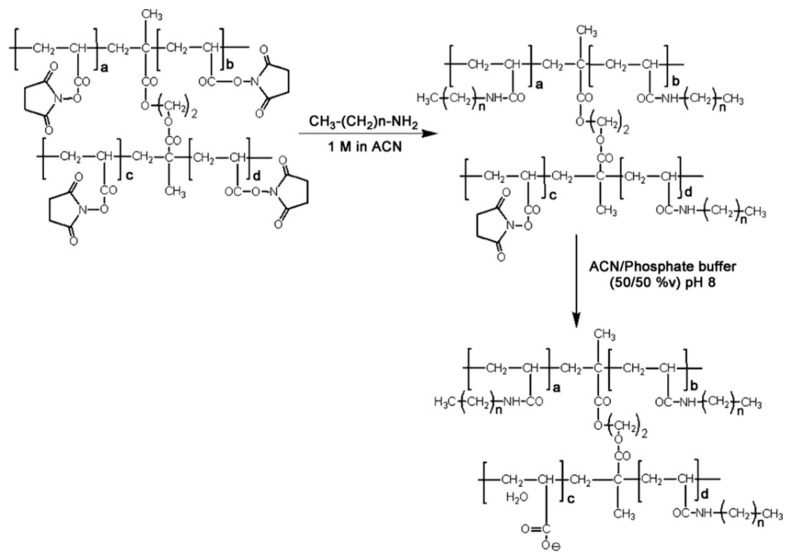
Reaction of functionalization with alkylamines (3 ≤ n ≤ 7) and hydrolysis of the porous methacrylic monolith having *N*-hydroxysuccinimide ester moieties attached to the polymeric matrix. Taken with permission from [25].

Guerrouache et al. [25] reported the study of functionalization of monolithic capillary column using *N*-acryloxysuccinimide (NAS) as a functional monomer in the template monolith and various alkylamine ligands were attached to the surface of the NAS-based monolith for RPC. The precursor monolith of poly(NAS-*co*-EDMA) was prepared with NAS and EDMA in toluene as porogen solvent via an in situ photopolymerization in the presence of AIBN. The obtained monolith was then subjected to PPF (see Scheme 16) with alkylamines by flushing the monoliths with alkylamine solutions for 30 min at a flow rate of 1 µL/min. The analysis of Raman Spectra was obtained before and after the PPF process to insure the grafting of the alkylamines to the surface of the monolith. The monolithic capillary columns were evaluated in terms of retention, selectivity and efficiency using alkylbenzenes and PAHs as solute probes in reversed-phase CEC (RP-CEC). The results of the electrochromatography were based, among other things, on the chemical structure of different alkyl chains in the monolith. 

The same research group continued their investigation on functionalizing monolithic capillary column using NAS and reported shortly after the above investigation, the preparation of hexylamine-functionalized poly(NAS-*co*-EDMA) and its ability to separate aromatic compounds and proteins in CEC [26]. It was demonstrated that the monolithic capillary columns followed the reversed-phase behavior. The hexylamine-functionalized poly(NAS-*co*-EDMA) were successfully able to separate alkylbenzenes, anilines, amino acids, phenols, organic acids and proteins.

Furthermore, the same group reported that the precursor poly(NAS-*co*-EDMA) can be functionalized with chiraly active ligands for chiral capillary chromatography [27]. Propargylamine is one of the alkylamines and was first attached to the surface of the monolith. Then, β-cyclodextrin was grafted to the functionalized poly(NAS-*co*-EDMA). The characterization of the monolith was obtained after each synthetic step by using for example, Raman spectroscopy. The enantioseparations of flavanone enantiomers were successfully obtained in both CEC and nano-LC. 

**Scheme 17:**
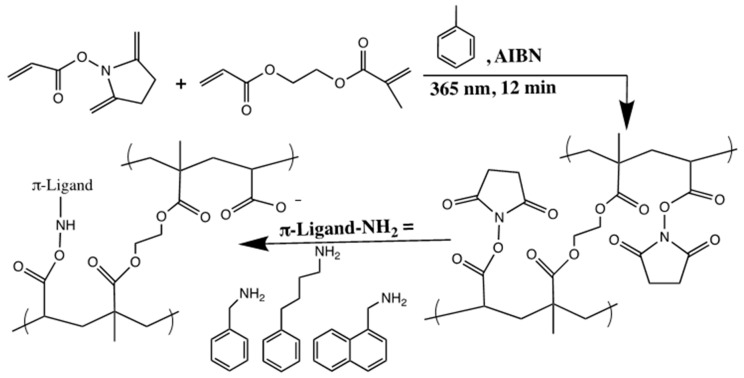
Schematic representation of the two-step synthetic pathway applied to prepare poly(*N*-acryloxysuccinimide (NAS)-*co*-EDMA) monolith functionalized with varied aromatic selectors: (i) NAS and EDMA are photopolymerized in the presence of toluene as porogen; (ii) aromatic amines are grafted on the surface of the macroporous monolith via nucleophilic substitution of *N*-hydroxysuccinimide esters. Residual activated esters lead, under well-defined conditions, to carboxylic groups enabling generation of electroosmotic flow (EOF). Adapted with modification [28].

In another study, the same group focused on functionalizing the surface of microporous monolithic capillary columns with varying aromatic ligands to be used in RP-CEC [28]. A mixture containing NAS and EDMA in toluene solution as porogen and in the presence of AIBN was prepared via in situ photopolymerization to obtain poly(NAS-*co*-EDMA). The next step was to graft the aromatic amines, such as benzylamine, phenylbutylamine and naphthylamine, individually on the surface of the precursor monolith by nucleophilic substitution of N-hydroxysuccinimide esters (see Scheme 17). The obtained functionalized poly(NAS-*co*-EDMA) monoliths were evaluated in terms of resolution, retention and selectivity. It was determined that the small change in the chemical structure of the aromatic selectors as ligands gave different chromatographic results. 

**Scheme 18:**
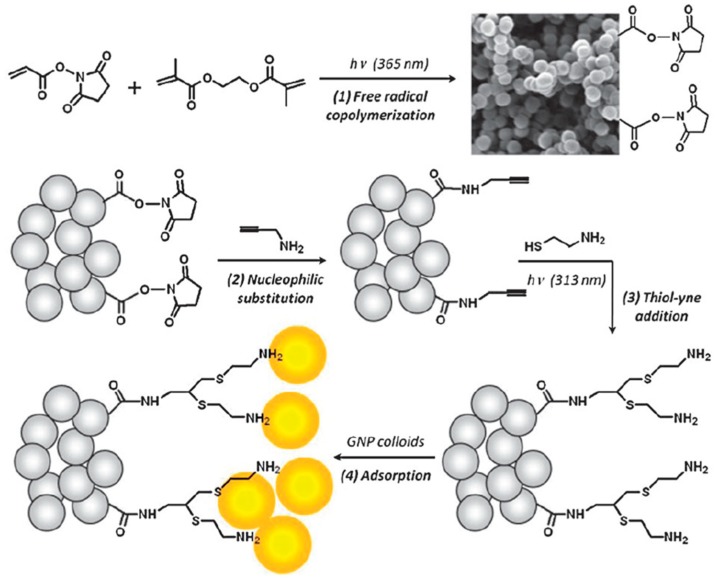
Reaction scheme for the surface immobilization of gold nanoparticles (GNPs) on the poly(NAS-*co*-EDMA) interface via free radical UV-initiated copolymerization, surface grafting of propargylamine and subsequent photo-initiated thiol-yne functionalization. Bis-cysteamine addition on alkyne units is assumed to be the major reaction mechanism. Taken with permission from [29].

The immobilization of gold nanoparticles to the surface of poly(NAS-*co*-EDMA) through the thiol-yne click chemistry was also developed [29]. The propargylamine was first attached to the poly(NAS-*co*-EDMA) monolith by nucleophilic substitution and then the cysteamine was grafted to the surface through thiol-yne addition reaction (see Scheme 18). Afterwards, the adsorption of gold nanoparticles to the monolithic surface was performed. This proposed method allowed the design of microdevices containing nanostructure and it can be used in microfluidic chip technology. 

**Scheme 19:**
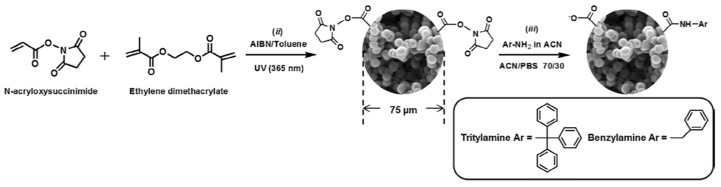
Schematic representation of two of the synthetic steps applied to prepare poly(NAS-*co*-EDMA) monolith functionalized with tritylamine or benzylamine: (ii) NAS and EDMA are photopolymerized in the presence of toluene as porogen and AIBN as initiator, (iii) aromatic amine is grafted on the surface of the macroporous monolith via nucleophilic substitution of N-hydroxysuccinimide esters. Residual activated esters lead, under well-defined conditions, to carboxylic groups enabling the generation of cathodic EOF. Taken with permission from [30].

**Scheme 20:**
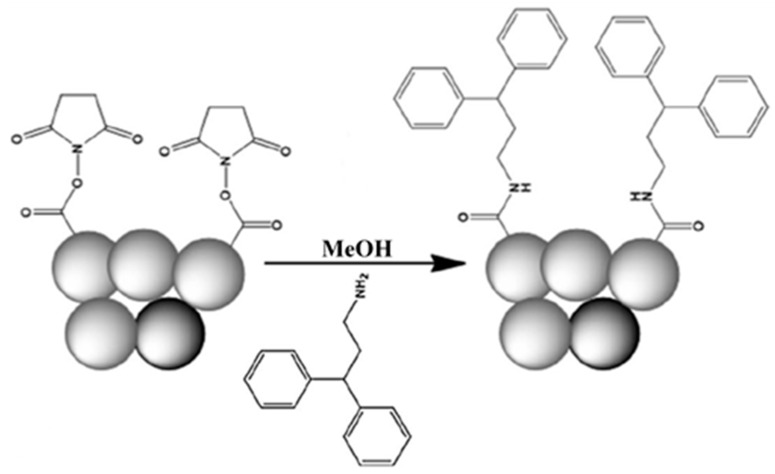
Schematic representation of the surface functionalization of poly(NAS-*co*-EDMA) monolith with 3,3-diphenylpropylamine. Adapted with modification [30].

In addition, a triphenylmethylamine-functionalized poly(NAS-*co*-EDMA) was prepared as new monolithic capillary column for RPC [30]. The generic monolith of poly(NAS-*co*-EDMA) was subjected to the post-polymerization modification with three ligands (i.e., tritylamine, 3,3-diphenylpropylamine, or benzylamine) as shown in Scheme 19 and Scheme 20. The monolithic columns thus obtained were evaluated with PAHs, phenols and anilines in terms of retention factor, selectivity, resolution and efficiency. 

**Scheme 21:**
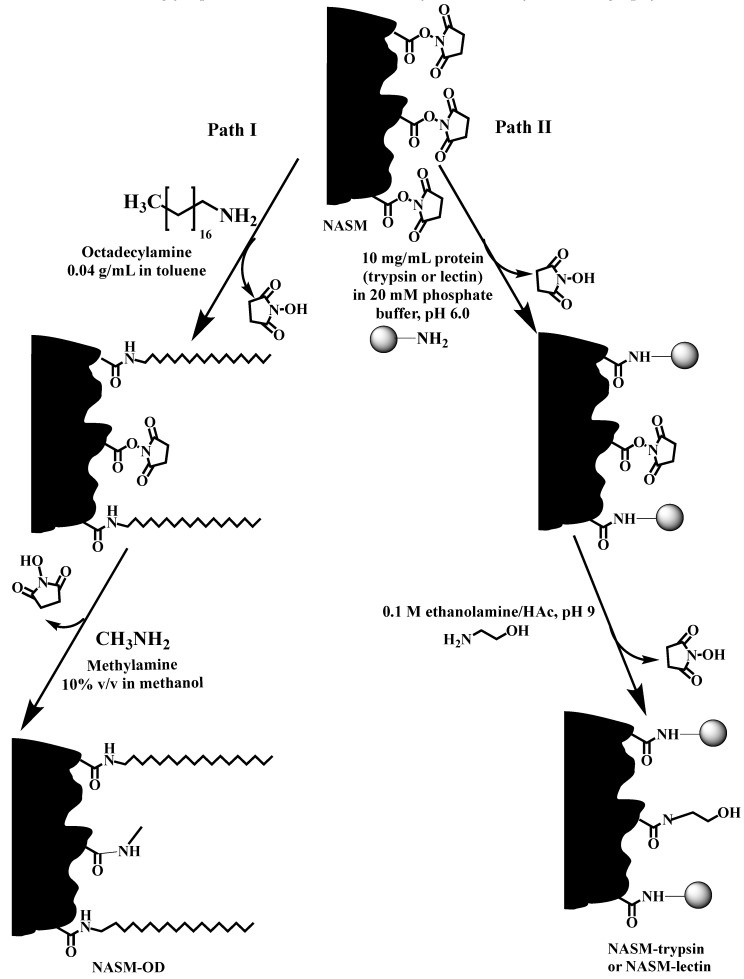
Post-polymerization modification of NASM with octadecyl and protein ligands. Path I: reaction of the surface succinimide groups of NASM with octadecylamine followed by scavenging the unreacted succinimide groups with methylamine; Path II: immobilization of protein (trypsin or lectin) on the surface succinimide groups of NASM followed by scavenging the unreacted succinimide groups of NASM with ethanolamine. Taken with permission from [31].

The preparation of a monolithic precursor and its PPF with alkyl ligands, trypsin and lectins for RPC, protein enzymatic digestion and lectin affinity chromatography, respectively, was recently reported (see Scheme 21) [31]. In this report, a precursor monolith consisting of poly(NAS-*co*-EDMA) (so-called NNASM) was synthesized by an in situ thermal polymerization in a narrow bore stainless-steel column. After the polymerization was completed, the obtained poly(NAS-*co*-EDMA) was modified with three types of ligands via the process of post-polymerization modification. Firstly, octadecyl ligands were attached to the surface of the poly(NAS-*co*-EDMA) monolith by passing octadecylamine through the column. This nonpolar monolith was used for the RPC separations of alkylbenzenes, PAHs and proteins yielding high resolution. Secondly, the surface of *N*-acryloxysuccinimide monolith (NASM) was immobilized with trypsin in order to use it for the online digestion of proteins. Thirdly, lectins immobilized on the surface of NASM was demonstrated in the selective isolation of glycoproteins from human serum by lectin affinity chromatography. 

### 2.6. PPF of Chloro Monoliths 

**Scheme 22:**
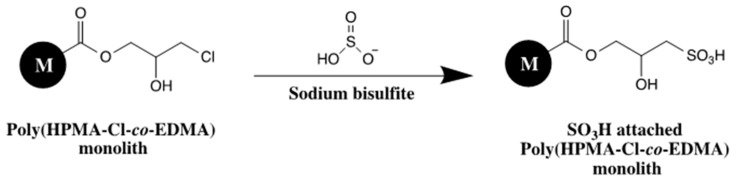
Scheme for grafting sulfonic acid functionality on poly(HPMA-Cl-*co*-EDMA) monolith. Adapted with modification [32].

Sulfonic acid functionalized poly(3-chloro-2-hydroxypropyl methacrylate (HPMA-Cl)-*co*-EDMA) was synthesized as new a monolith for CEC applications [32]. The template monolith was first obtained via in situ polymerization of HPMA-Cl as monomer and EDMA as crosslinker in the presence of AIBN. The porogen mixture used was isopropanol and *n*-decanol. After that, poly(HPMA-Cl-*co*-EDMA) was subjected to PPF with NaHSO_3_ to achieve strong sulfonated monolith, see Scheme 22. The template monolith can be easily modified with different ligands such as, anionic, cationic or neutral groups to have different CEC applications. The proposed monolithic capillary column could successfully separate phenol derivatives, alkylbenzenes and benzoic acids. 

**Scheme 23:**
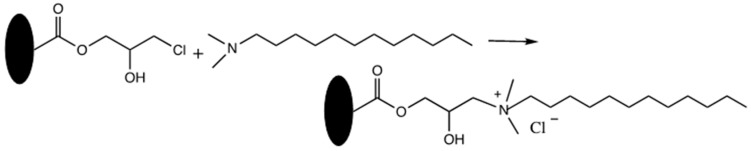
Schematic reaction of a new poly(3-chloro-2-hydroxypropyl methacrylate (HPMA-Cl)-*co*-EDMA) monolith with DMDA. Adapted with modification [33].

A new mixed-mode monolithic capillary column was successfully prepared and evaluated in nano-LC [33]. First, the poly(HPMA-Cl-*co*-EDMA) was synthesized by in situ polymerization. Next, the resulting monolith was modified with *N*,*N*-dimethyl-*N*-dodecylamine to achieve a mixed-mode monolith (i.e., anion-exchange and hydrophobic), see Scheme 23. The separation of alkylbenzenes, phenolic compounds and inorganic anions was successfully resolved due to the multiple interactions including hydrophobic, electrostatic and anion exchange. The column efficiency was found to be up to 23,000 plates/m for the separation of inorganic anions. 

**Scheme 24:**
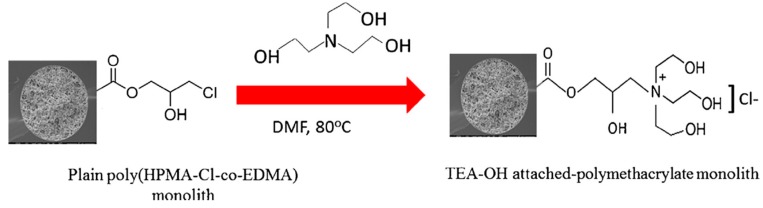
Schematic of the derivatization of poly(HPMA-Cl-*co*-EDMA) monolith with triethanolamine solution (TEA-OH). Taken with permission from [34].

Kip et al. [34] reported the preparation of a monolithic capillary column and its surface modification for HILIC mode in nano-LC. The poly(HPMA-Cl-*co*-EDMA) was prepared in capillary by in situ radical polymerization. After the polymerization was completed, a triethanolamine solution (TEA-OH) was passed through the monolithic capillary column in order to increase the hydrophilicity of the monolith, see Scheme 24. The separation of nucleotides, nucleosides and benzoic acid derivatives was successfully resolved with estimated plate heights up to 20 µm. 

In another study, the poly(HPMA-Cl-*co*-EDMA) monolith was subjected to the post-polymerization modification with polyethyleneimine obtaining cationic stationary phase for reversed phase and hydrophilic interaction in CEC [35]. The obtained monolithic column was successfully used for the separation of alkylbenzenes, phenols, PAHs and nucleosides. 

### 2.7. PPF of Hydroxy Monoliths

**Scheme 25:**
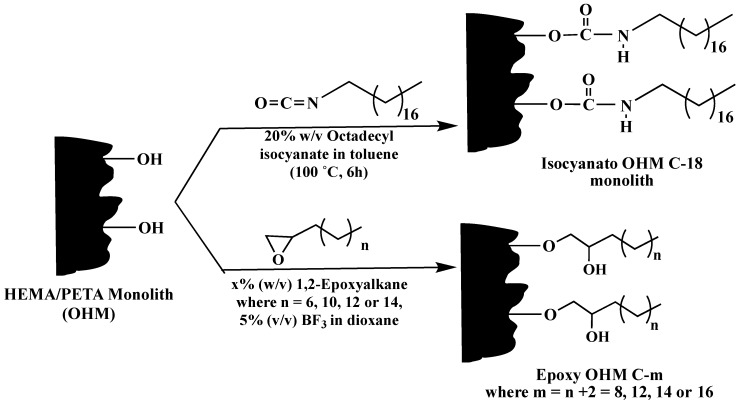
Schematic of the reaction of the precursor poly(HEMA-*co*-PETA) monolith (i.e., OHM) with 1,2-epoxyalkanes at varying alkyl chain length and with octadecyl isocyanate. The post-polymerization reaction was carried out for 6 h. Taken with permission from [36].

A novel poly(hydroxyethyl methacrylate (HEMA)-*co*-pentaerythritol triacrylate (PETA)) monolithic capillary column was prepared and subjected to PPF with epoxy alkane, octadecyl isocyanate and epoxy biphenyl for reversed phase CEC [36,37]. The template monolith referred to as hydroxy monolith (OHM) capillary column was synthesized via in situ thermal polymerization of a mixture containing HEMA as the functional monomer and PETA as cross-linker in the presence of cyclohexanol and 1-dodecanol as binary porogen to obtain neutral monolith. The obtained OHM capillary column was then grafted with epoxy alkanes at varying alkyl chain lengths to have epoxy OHM C-m, where m = 8, 12, 14 and 16, see Scheme 25. The same template monolith was also grafted with octadecyl isocyanate to obtain Isocyanato OHM C-18 (Scheme 25). The study demonstrated that changing the alkyl chain length in the monolith could change the column phase ratio and the solute distribution constant. Small solutes at different polarity have been tested with these capillary columns and showed successful separation performance.

**Scheme 26:**
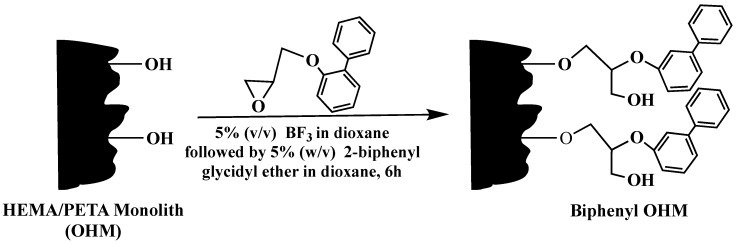
Post-polymerization modification of the poly(HEMA-*co*-PETA) precursor monolith with 2-biphenylglycidyl ether to yield the Biphenyl OHM HPLC column and the Biphenyl OHM capillary column. The reaction was performed at 20 ˚C for 6 h. Taken with permission from [37].

Moreover, the poly(HEMA-*co*-PETA) monolith was subjected to PPF with epoxy biphenyl yielding biphenyl OHM capillary column [37], see Scheme 26. The obtained monolithic column exhibited hydrophobic interactions and π−π interactions with various solutes. The Biphenyl OHM capillary has successfully separated different compounds, such as anilines derivatives, phenols and phenoxy acid herbicides with good reproducibility. 

**Scheme 27:**
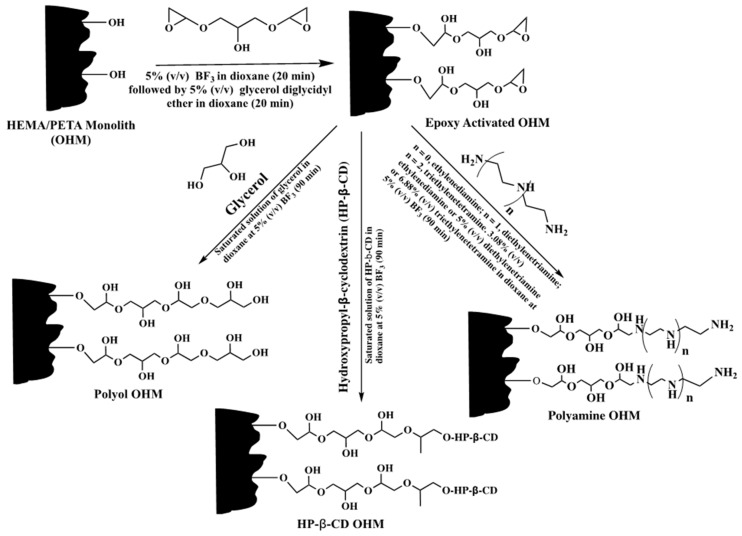
Reaction schemes of post-polymerization modification of poly(HEMA-*co*-PETA) precursor monolith for making polyol, polyamine and hydroxypropyl-β-CD (HP-β-CD) monoliths. Taken with permission from [38].

The poly(HEMA-*co*-PETA) monolith, on the other hand, was activated with glycerol diglycidyl ether to have epoxy activated OHM for CEC, see Scheme 27 [38]. The activated monolithic capillary column was then subjected to PPF with either glycerol or polyamines to obtain polyol OHM and polyamine OHM columns, respectively. These resulted columns were used in HILIC. In addition, the epoxy activated OHM was also grafted with hydroxypropyl-β-CD (HP-β-CD) to yield HP-β-CD OHM for enantioseparation. These columns exhibited good separation performance and good reproducibility. 

### 2.8. PPF of Carboxy Monoliths

**Scheme 28:**
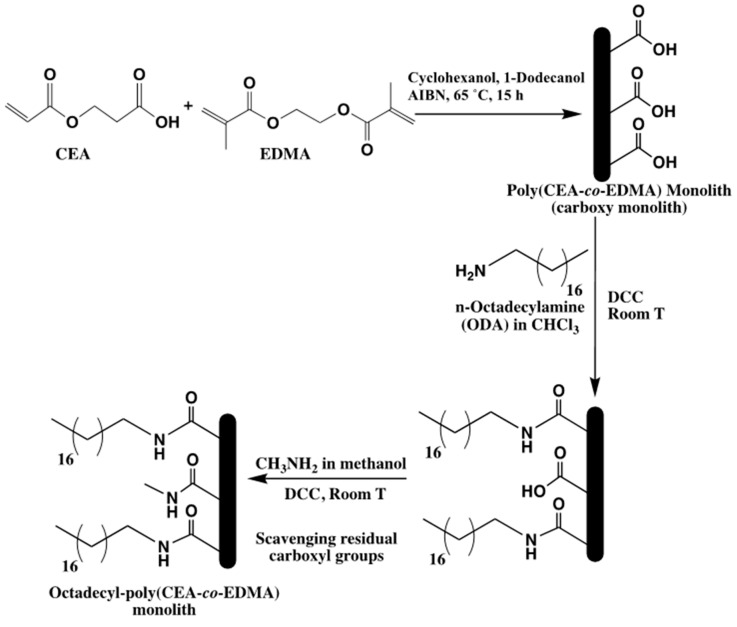
Schematic for the preparation of poly(2-carboxyethyl acrylate (CEA)-*co*-EDMA) monolith and its post-polymerization modification with octadecylamine (ODA) ligands. Taken with permission from [39].

A new precursor monolithic capillary column was prepared by the in situ co-polymerization of 2-carboxyethyl acrylate (CEA) and EDMA in a 300 μm i.d. fused silica capillary yielding a poly(CEA-*co*-EDMA) monolithic column [39]. This carboxy monolith precursor with its surface carboxyl groups was subsequently subjected to PPF with octadecylamine (ODA) via a carbodiimide conjugation reaction in the presence of an organic soluble carbodiimide, *N,N′*-dicyclohexylcarbodiimide (DCC). see Scheme 28. The carbodiimide with its zero-length carboxyl-to-amine coupling ability works by activating the surface carboxyl groups of the monolith for direct reaction with the primary amines of ODA via amide bond formation. This reaction, which is applied for the first time in monolithic column fabrication, yielded the octadecyl-poly(CEA-*co*-EDMA) monolithic capillary column for use in reversed phase capillary LC. This octadecyl column was evaluated for its RPC retention property with alkylbenzenes (ABs) and alkyl phenyl ketones (APKs). The methylene group selectivity (α_CH2_) in the particular homologs was about the same for both ABs and APKs (α_CH2_ = 1.6) using the same mobile phase composition. This may indicate that the retention mechanism is the same for both homologous series. The retention behaviors of polyaromatic hydrocarbons and chlorophenol compounds were also examined on the octadecyl capillary column. The study showed good retention and selectivity of octadecyl-poly(CEA-*co*-EDMA) monolithic capillary column toward the various solutes under investigation. In addition, the column showed good permeability and reproducibility. 

**Scheme 29:**
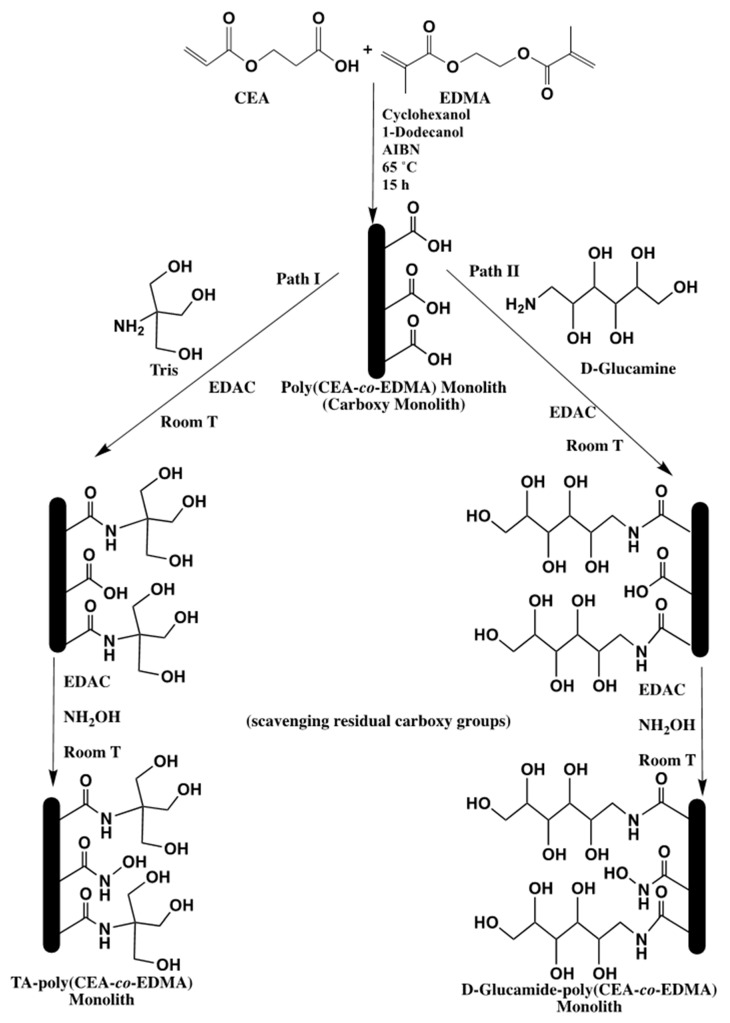
Schematic diagram showing the preparation of poly(CEA-*co*-EDMA) monolith and its PPF with (I) Tris and (II) _D_-Glucamine. Taken with permission from [40].

The same precursor monolithic capillary column (carboxy monolith), namely poly(CEA-*co*-EDMA) was subjected also to PPF with Tris and D-Glucamine in the presence of *N*-(3-dimethylamineopropyl)-*N*′-ethylcarbodiimide hydrochloride (EDAC), a water soluble carbodiimide for the use in HILIC (see Scheme 29) [40]. Similarly, to DDC, the EDAC with its zero-length carboxyl-to-amine coupling ability works by activating the surface carboxyl groups of the monolith for direct reaction with the primary amines of Tris and D-Glucamine via amide bond formation. The Tris-amide (TA)- and D-Glucamide-poly(CEA-*co*-EDMA) monolithic capillary columns were evaluated using neutral and charged polar analytes, such as phenolic acids, nucleic acid bases and nucleosides. The difference in polarity between these monolithic capillary columns is noticeable in terms of the numbers of hydroxyl group per ligands, and therefore, the D-Glucamide-poly(CEA-*co*-EDMA) monolith bearing 5 hydroxyl groups per ligand could separate more polar species compared to the TA-poly(CEA-*co*-EDMA) monolith which has 3 hydroxyl groups per ligand. Solute retention on both types of HILIC columns were reproducible from run-to-run, day-to-day and column-to-column. 

### 2.9. PPF of Nanoparticles Incorporated Monoliths

**Scheme 30:**
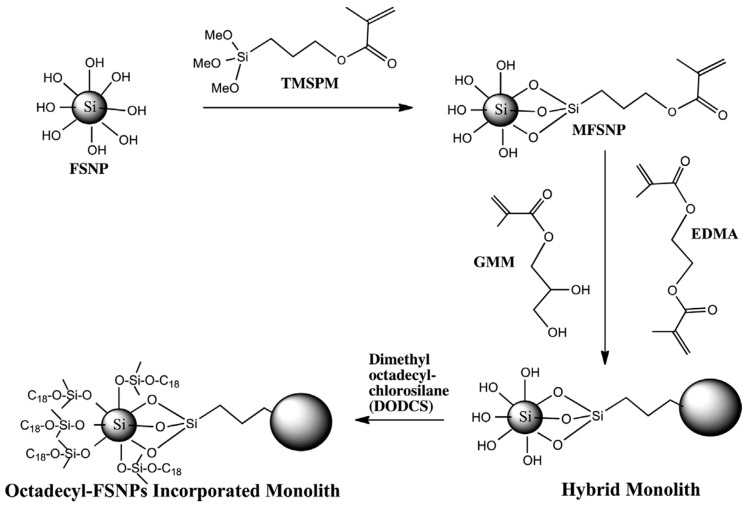
Schematic diagram of the synthesis of the glyceryl monomethacrylate (GMM)/EDMA/methacryloyl fumed silica nanoparticle (MFSNP) monolith followed by grafting the surface of the hybrid monolith with octadecyl ligands. Taken with permission from [41].

Recently, Aydoğan and El Rassi reported polymethacrylate based monolithic columns incorporated with surface modified fumed silica nanoparticles (FSNPs) for use in reversed phase chromatography (RPC) of small solutes and proteins. First, FSNPs were modified with 3-(trimethoxysilyl)propylmethacrylate (TMSPM) to yield the “hybrid” methacryloyl fumed silica nanoparticle (MFSNP) monomer [41]. The resulting MFSNP was then mixed with glyceryl monomethacrylate (GMM) and EDMA in a binary porogenic solvent composed of cyclohexanol and dodecanol, and the in situ copolymerization of MFSNP, GMM and EDMA was performed in a stainless steel column of 4.6 mm i.d. (see Scheme 30). The hybrid monolith thus obtained was subjected to PPF that involved the grafting of the free silanols with octadecyl ligands by perfusing the hybrid monolithic column with a solution of 4 % *w*/*v* of dimethyloctadecylchlorosilane (DODCS) in toluene while the column was maintained at 110 ˚C for 6 h (in a heated HPLC oven). One of the originalities of this study was to demonstrate MFSNP as a novel derivatized “hybrid monomer” in making RPC monolithic columns with surface bound octadecyl ligands (Scheme 30). In this respect, the RPC behavior of the monolithic column with “covalently” incorporated FNSPs having surface grafted octadecyl ligands was evaluated with alkylbenzenes, aniline derivatives and phenolic compounds. The results showed that the hybrid poly(GMA-MFSNP-EDMA) having surface bound octadecyl ligands exhibited hydrophobic interactions under reversed phase elution conditions. Furthermore, six standard proteins were baseline separated on the column using a 10 min linear gradient elution at increasing ACN concentration in the mobile phase at a flow rate of 1.0 mL/min. 

**Scheme 31:**
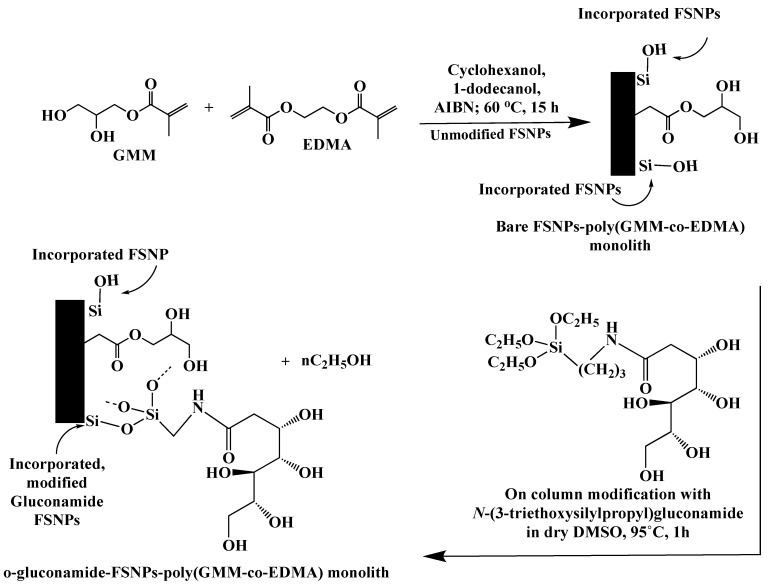
Scheme of the fabrication of the poly(GMM-*co*-EDMA) monolithic columns including incorporated bare FSNPs-poly(GMM-*co*-EDMA) monolith and monolith with incorporated on-column modified gluconamide FSNPs (o-gluconamide-FSNPs- poly(GMM-co-EDMA)). Adapted with modification [42].

In another development involving PPF of nanoparticle incorporated monoliths, Ganewatta and El Rassi [42] reported the preparation and characterization of monolithic capillary columns with incorporated bare fumed silica nanoparticles (FSNPs) and surface coated gluconamide FSNPs and their subsequent use in hydrophilic interaction capillary electrochromatography (HI-CEC) of small relatively polar solutes. The monolithic support was based on the in situ polymerization of GMM and EDMA in the presence of FSNPs yielding the poly(GMM-*co*-EDMA) monolith with incorporated FSNPs (FSNPs-poly(GMM-*co*-EDMA) monolith), which was subsequently subjected to PPF with *N*-(3-triethoxysilylpropyl)gluconamide in an on-column approach that resulted in o-gluconamide-FSNPs-poly(GMM-*co*-EDMA) monolith see Scheme 31. This approach yielded a better CEC performance than the monolithic column with incorporated FSNPs that were treated pre-column with *N*-(3-triethoxysilylpropyl)gluconamide (p-gluconamide-FSNPs). It is believed that while the p-gluconamide-FSNPs was coated by an oligosiloxane gluconamide layer as was revealed by thermogravimetric analysis, the o-gluconamide-FSNPs are thought to be covered with a monomeric layer of gluconamide ligands as was manifested by the higher plate number obtained on the latter than on the former gluconamide-FSNPs incorporated monolithic columns. In the on-column modification process of FSNPs, the reaction was performed in a closed system whereby atmospheric water vapor are not available to cause the polymerization of the trifunctional organosilane *N*-(3-triethoxysilylpropyl)gluconamide. Additionally, the fact that the o-gluconamide-FSNPs incorporated monoliths were made from bare-FSNPs incorporated monoliths may indicate that the bare FSNPs were better dispersed into the monolithic matrix than the p-gluconamide-FSNPs, a condition that might have further contributed to the lower plate count obtained on p-gluconamide- than o-gluconamide-FSNPs incorporated monolithic columns. Overall, o-gluconamide-FSNPs stationary phases and to a lesser extent bare-FSNPs stationary phases proved useful in HI-CEC of small polar solutes, including DMF, formamide, thiourea, some phenols and nucleobases. 

### 2.10. Miscellaneous

**Figure 2:**
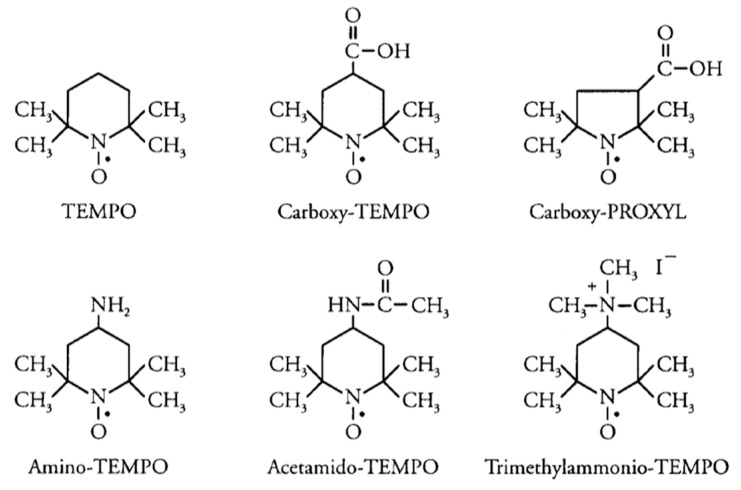
Porous poly(styrene-co-divinylbenzene) monoliths with controlled pore size distribution have been prepared using new types of stable free radical (SFR) initiators in HPLC [43].

Porous poly(styrene-*co-*divinylbenzene) monoliths with controlled pore size distribution have been prepared using new types of stable free radical (SFR) initiators in HPLC [43]. The origin monolith was prepared via in situ polymerization of a mixture consisting of divinylbenzene and styrene mixed in 1-decanol and poly(ethylene glycol) as porogenic solution in the presence of benzoyl peroxide as initiator and different types of SFR, see the following structures (Figure 2). The pore size distribution was studied for all types of SFR. The study demonstrated that the carboxy-2,2,6,6-tetramethylpiperidinyl-1-oxy (carboxy-TEMPO) and 3-carboxy-2,2,5,5-tetramethylpyrrolidinyl-1-oxy (carboxy-PROXYL) improved the permeability of the monolith compared with other SFR. The poly(styrene-*co-*divinylbenzene) monolith using carboxy functional SFRs was grafted with 2-hydroxyethyl methacrylate and also with 3-sulfopropyl methacrylate individually to obtain a variety of monolithic surfaces. 

The preparation of a continuous-bed column and its PPF with macrocyclic antibiotic as chiral selector was proposed for use in CEC [44]. The continuous bed capillary column was prepared first via in situ polymerization of a mixture containing *N*-(hydroxymethyl)acrylamide, piperazine diacylamine, *N*,*N*’-diallyltetradiamide, *N*,*N*,*N*’,*N*’-tetramethylethylenediamine, ammonium persulfate and vinyl sulfonic acid. Then, the obtained column was grafted with vancomycin by a reductive amination process. The enantioseparations of thalidomide, bupivacaine and warfarin were successfully obtained using the vancomycin based nonparticulate bed. 

**Scheme 32:**
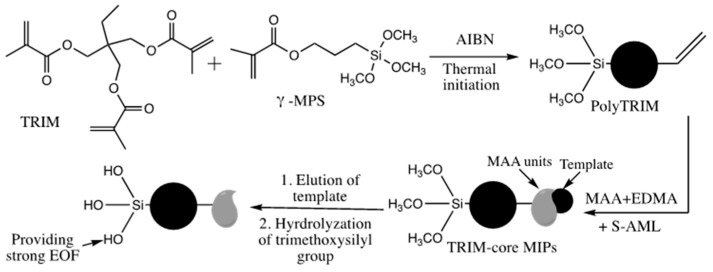
Schematic representations of the preparation of grafted MIP capillary with (S)-amlodipine (S-AML) imprints. Adapted with modification [45].

A molecularly imprinted polymer (MIP) coating was grafted on a poly(TRIM) monolithic capillary column for use in chiral separation by CEC [45]. The origin monolith was prepared via in situ polymerization of a mixture containing TRIM in the presence of a binary porogen under thermal initiator condition. The grafted MIP was obtained by passing a mixture of template, methacrylic acid and EDMA as monomers, porogen and thermal initiator into the TRIM monolithic column, see Scheme 32. Then the capillary was incubated in water bath at a temperature of 53 °C until polymerization. The proposed monolith provided high resolution for amlodipine enantiomers separation up to 7.99.

## 3. Concluding Remarks

Briefly, this article has reviewed for the first time the various grafting reactions that have been performed by PPF on polymer-based monoliths used in liquid phase separation techniques, namely, HPLC, nano- and micro-LC and CEC. In addition, this article highlighted the different separations carried out on the monolithic columns. Thus, this review article can be viewed as a “central review bank” in the various aspects of monolithic precursors and their PPF with a variety of chromatographic ligands, which will facilitate the design of separations in the separation sciences in particular and the life sciences in general.
